# Sustainable synthesis of calcium propionate from postbiotic propionic acid produced by Schleiferilactobacillus harbinensis–Pichia kudriavzevii co-culture using alternative calcium sources: Physicochemical characterization and feed additive potential

**DOI:** 10.14202/vetworld.2026.3932-3943

**Published:** 2026-09-11

**Authors:** Yetti Marlida, Fauzia Agustin, Lili Anggraini, Harnentis Harnentis, Laily Rinda Ardani, Thelsa Anggun Bagaskarel

**Affiliations:** 1 Department of Animal Nutrition, Faculty of Animal Science, Universitas Andalas, Padang, West Sumatra 25163, Indonesia.; 2 Research Organization for Agriculture and Food, National Research and Innovation Agency (BRIN), Bogor 16911, Indonesia.; 3 Doctoral Program, Faculty of Animal Science, Universitas Andalas, Padang, West Sumatra 25163, Indonesia.

**Keywords:** alternative calcium sources, calcium propionate, circular bioeconomy, eggshell waste, feed additive, Pichia kudriavzevii, postbiotic propionic acid, Schleiferilactobacillus harbinensis

## Abstract

**Background and Aim:**

Calcium propionate is a multifunctional feed additive widely used to improve energy metabolism, prevent metabolic disorders, and enhance feed preservation in livestock production. However, commercial calcium propionate is predominantly synthesized from petrochemical-derived propionic acid, raising concerns regarding sustainability and resource dependence. This study aimed to synthesize calcium propionate using bio-derived propionic acid produced by a co-culture of *Schleiferilactobacillus* *harbinensis* and *Pichia kudriavzevii*, compare four alternative calcium sources, and identify the most suitable material for producing high-quality calcium propionate through comprehensive physicochemical characterization.

**Materials and Methods:**

Bio-derived propionic acid obtained from an optimized microbial co-culture fermentation was reacted with eggshell flour, shellfish meal, bone meal, and indigenous limestone flour under optimized synthesis conditions (200-mesh particle size, 15% propionic acid, solid-to-liquid ratio of 0.14:1, 80°C, and 2.5 h). The synthesized products were characterized using scanning electron microscopy coupled with energy-dispersive spectroscopy (SEM–EDS), Fourier transform infrared spectroscopy (FTIR), and X-ray diffraction (XRD) to evaluate morphology, elemental composition, functional groups, and crystalline structure.

**Results:**

The calcium source markedly influenced the physicochemical characteristics of the synthesized calcium propionate. Eggshell-derived calcium propionate exhibited the most porous and heterogeneous microstructure, indicating a greater apparent surface area than products synthesized from the other calcium sources. EDS analysis confirmed that calcium, carbon, and oxygen were the predominant elements in all products, while the eggshell-derived product showed the most homogeneous elemental composition with fewer detectable impurities. FTIR spectra confirmed successful calcium propionate formation in all treatments through characteristic carboxylate absorption bands, whereas the eggshell-derived product exhibited sharper and better-defined absorption peaks, indicating greater chemical uniformity. XRD analysis further demonstrated a more ordered crystalline structure and fewer secondary mineral phases in the eggshell-derived product than in the shellfish meal-, bone meal-, and limestone-derived products. Collectively, SEM–EDS, FTIR, and XRD consistently identified eggshell flour as the most suitable calcium precursor for producing high-quality calcium propionate.

**Conclusion:**

Eggshell flour is a promising sustainable calcium source for producing high-quality calcium propionate from bio-derived propionic acid. Integrating postbiotic propionic acid production with eggshell waste valorization provides an environmentally friendly approach for manufacturing value-added feed additives while supporting circular bioeconomy principles and sustainable livestock production.

## INTRODUCTION

Calcium propionate is widely recognized as an effective and multifunctional feed additive in dairy production systems, particularly during the periparturient period. It helps mitigate metabolic disorders, including negative energy balance (NEB), hypocalcemia, and ketosis, which are common challenges during early lactation in dairy cows [1]. As a source of both calcium and propionic acid, calcium propionate contributes substantially to ruminal gluconeogenesis, supplying approximately 60%–74% of the glucose precursors required for energy metabolism in ruminants [2]. Consequently, calcium propionate supplementation has been shown to improve feed intake, milk production, and overall metabolic health in dairy cattle [1]. In addition to its nutritional benefits, calcium propionate possesses antimicrobial activity that inhibits mold growth and reduces mycotoxin contamination, making it an effective feed preservative [3]. Despite these advantages, commercial calcium propionate is predominantly manufactured through conventional chemical synthesis using petrochemical-derived propionic acid. This production route raises concerns regarding dependence on non-renewable resources, environmental sustainability, and long-term production costs. Consequently, increasing demand for environmentally sustainable livestock production has stimulated considerable interest in developing bio-based alternatives. Among these, microbial production of postbiotic propionic acid represents a promising approach because it utilizes renewable biological resources while reducing the environmental footprint associated with conventional chemical synthesis.

Recent advances in industrial fermentation have demonstrated the advantages of mixed microbial cultures for improving metabolite production through complementary metabolic interactions. Co-culture systems involving *Schleiferilactobacillus* *harbinensis* and *Pichia kudriavzevii* exhibit synergistic relationships that enhance substrate utilization efficiency, fermentation stability, and organic acid production [4]. In the present study, postbiotic propionic acid produced by this bacterial–yeast co-culture was used as the precursor for calcium propionate synthesis, representing the first reported application of this microbial consortium for producing bio-based calcium propionate. However, investigations into optimizing and scaling up postbiotic propionic acid production using this co-culture remain limited. Beyond upstream fermentation, downstream synthesis of calcium propionate offers further opportunities to improve sustainability. Conventionally, calcium propionate is synthesized by neutralizing propionic acid with calcium-based compounds, typically calcium carbonate or calcium hydroxide [1]. Recently, alternative calcium sources derived from agro-industrial by-products, including eggshells, shellfish waste, bone meal, and natural limestone, have attracted increasing attention because of their high calcium content, low cost, widespread availability, and potential contribution to waste valorization. Nevertheless, systematic comparisons of these alternative calcium sources in terms of synthesis efficiency, product quality, and physicochemical characteristics remain scarce. Furthermore, synthesis variables such as particle size, propionic acid concentration, solid-to-liquid ratio, reaction temperature, and reaction time substantially influence calcium propionate quality and yield. Although previous studies have investigated these parameters individually, an integrated approach combining optimized bio-derived propionic acid production with sustainable downstream calcium propionate synthesis has not yet been comprehensively explored.

Although previous studies have reported microbial production of propionic acid and the synthesis of calcium propionate from conventional chemical precursors, important knowledge gaps remain. To date, no study has comprehensively integrated postbiotic propionic acid produced by an *S. harbinensis–P. kudriavzevii* co-culture with the sustainable synthesis of calcium propionate using multiple alternative calcium sources derived from agro-industrial waste. Moreover, comparative information on how different waste-derived calcium precursors affect the physicochemical properties, elemental composition, crystalline structure, and overall quality of the synthesized calcium propionate remains limited. This lack of comparative evidence limits identification of the most suitable renewable calcium source for producing high-quality calcium propionate while supporting waste valorization and circular bioeconomy strategies. Therefore, an integrated evaluation encompassing both renewable propionic acid production and sustainable calcium source selection is needed to establish an environmentally friendly manufacturing approach for value-added livestock feed additives.

This study aimed to synthesize calcium propionate from bio-derived propionic acid produced by a co-culture of *S. harbinensis* and *P. kudriavzevii*, to compare the suitability of four alternative calcium sources (eggshell flour, shellfish meal, bone meal, and indigenous limestone flour) for calcium propionate synthesis, and to comprehensively characterize the synthesized products using scanning electron microscopy coupled with energy-dispersive spectroscopy (SEM–EDS), Fourier transform infrared spectroscopy (FTIR), and X-ray diffraction (XRD). The study further sought to identify the most suitable sustainable calcium precursor capable of producing high-quality calcium propionate with desirable physicochemical characteristics for potential application as an environmentally friendly livestock feed additive.

## MATERIALS AND METHODS

### Ethical approval

This study did not require approval from an Institutional Animal Care and Use Committee because no live animals underwent experimental procedures. Rumen fluid was collected from apparently healthy goats immediately after routine commercial slaughter at a licensed local slaughterhouse. Sample collection was performed postmortem without any additional animal handling or intervention for research purposes. All procedures involving sample collection, transportation, microbial cultivation, and laboratory analyses complied with institutional biosafety and laboratory hygiene regulations. Laboratory procedures involving microorganisms were conducted under biosafety level 2 conditions to ensure personnel safety and prevent environmental contamination.

### Study period and location

The study was conducted from June to December 2025 at the Feed Technology Laboratory, Faculty of Animal Science, Universitas Andalas, Padang, West Sumatra, Indonesia. Production of postbiotic propionic acid, calcium propionate synthesis, and physicochemical characterization were performed at the same institution. 

### Study design

This experimental laboratory study evaluated the synthesis of calcium propionate from bio-derived propionic acid produced through microbial co-culture fermentation. Four alternative calcium sources were investigated: eggshell flour (P1), shellfish flour (P2), bone meal (P3), and indigenous limestone flour (P4). Calcium propionate synthesized from each calcium source was comparatively characterized using SEM–EDS, FTIR, XRD, and thermogravimetric analysis (TGA) to evaluate differences in morphology, elemental composition, functional groups, crystalline structure, and thermal stability.

### Microorganisms and inoculum preparation

The microbial strains used in this study were *S. harbinensis* and *P. kudriavzevii*, obtained from the Feed Technology Laboratory culture collection, Universitas Andalas, Padang, Indonesia. These microorganisms were selected because of their reported synergistic interactions in co-culture systems that enhance substrate utilization efficiency and organic acid production [4, 5].

Each strain was activated separately in an appropriate growth medium under controlled conditions before co-cultivation. Stock cultures were preserved at −80°C in their respective media supplemented with 20% (v/v) glycerol. Before experimentation, *S. harbinensis* was revived in De Man, Rogosa, and Sharpe (Merck, Darmstadt, Germany) broth and incubated anaerobically at 37°C for 24–48 h until the mid-logarithmic growth phase (OD₆₀₀ ≈ 0.8). *P. kudriavzevii* was cultured in Yeast extract–peptone–dextrose (Sigma-Aldrich, Merck, Darmstadt, Germany) broth at 35°C–37°C for 24–48 h until the exponential growth phase was reached. The inoculum density of each microorganism was standardized to ensure reproducibility and optimal fermentation performance, as recommended for mixed-culture fermentation systems [6].

### Fermentation process

Bio-derived propionic acid used in this study was obtained from a previously optimized co-culture fermentation of *S. harbinensis* and *P. kudriavzevii*, as described by Marlida *et al.* [4]. This study focused on using the resulting postbiotic propionic acid for calcium propionate synthesis and physicochemical characterization.

Propionic acid production was carried out using a previously optimized co-culture fermentation system. The fermentation medium composition, pH, incubation temperature, and inoculum ratio were adopted from previously optimized conditions because these variables significantly influence organic acid production [4]. Fermentation was performed under anaerobic or microaerophilic conditions favorable for propionic acid biosynthesis [7]. Samples were collected periodically to monitor propionic acid concentration, substrate utilization, and microbial growth throughout fermentation.

Based on the previous optimization study, fermentation was conducted under low carbon availability (0.1 g/L), high nitrogen concentration (15 g/L), and a 72 h incubation period. A 1:1 inoculum ratio with a total inoculum level of 4%, an initial pH of 7.0, and a forage-to-concentrate substrate ratio of 60:40 were used [4, 6]. These optimized conditions were selected because they critically affect microbial metabolic activity, fermentation efficiency, propionic acid production, and overall process productivity [8].

### Calcium propionate synthesis

**Raw materials:** The calcium sources evaluated in this study were eggshell flour, shellfish flour, bone meal, and indigenous limestone flour. These materials were selected for their high calcium content, low cost, wide availability, and potential as sustainable alternatives to commercially refined calcium sources.

Before synthesis, eggshells and shellfish shells were thoroughly washed with distilled water to remove adhering organic matter, air-dried, oven-dried to constant weight, ground into fine powder, and passed through a 200-mesh sieve. Bone meal and indigenous limestone were similarly dried, milled, and sieved to obtain a uniform particle size before use as calcium precursors.

**Experimental treatments:** Four calcium source treatments were evaluated: eggshell flour (P1), shellfish flour (P2), bone meal (P3), and indigenous limestone flour (P4). The synthesized calcium propionate products were comparatively characterized using SEM–EDS, FTIR, XRD, and TGA to evaluate differences in morphology, elemental composition, functional groups, crystalline structure, and thermal stability.

**Synthesis procedure:** Calcium propionate was synthesized by reacting bio-derived propionic acid with each calcium source under optimized reaction conditions reported previously [9]. The synthesis was performed using a particle size of 200 mesh, a propionic acid concentration of 15%, a solid-to-liquid ratio of 0.14:1, a reaction temperature of 80°C, and a reaction time of 2.5 h.

After the reaction was complete, the resulting slurry was filtered and washed thoroughly with distilled water to remove residual reactants. The recovered product was subsequently dried before physicochemical characterization.

### Product characterization

The synthesized calcium propionate was characterized using complementary analytical techniques to evaluate its physicochemical properties.

FTIR analysis was performed using an IRTracer-100 spectrometer (Shimadzu Corporation, Kyoto, Japan) to identify functional groups and confirm the chemical structure of the synthesized products [10]. XRD analysis was conducted using a D8 Advance diffractometer (Bruker AXS GmbH, Karlsruhe, Germany) to determine crystalline structure and phase composition [11]. SEM–EDS analysis was performed using a JSM-6510LV scanning electron microscope (JEOL Ltd., Tokyo, Japan) to evaluate surface morphology and elemental composition [12]. TGA was performed using a DTG-60H thermal analyzer (Shimadzu Corporation, Kyoto, Japan) to assess thermal stability and compositional characteristics of the synthesized products [13]. These complementary analytical techniques are widely employed for comprehensive physicochemical characterization and quality assessment of calcium-based materials.

### Statistical analysis

This study primarily involved qualitative physicochemical characterization of calcium propionate synthesized from four different calcium sources. Surface morphology, elemental composition, functional groups, crystalline structure, and thermal stability were comparatively evaluated using SEM–EDS, FTIR, XRD, and TGA. Because the study did not generate replicated quantitative response variables suitable for statistical hypothesis testing, inferential statistical analyses were not performed. Instead, comparative interpretation of the analytical findings was undertaken to identify the calcium source that produced calcium propionate with the most favorable physicochemical characteristics.

## RESULTS AND DISCUSSION

### Morphological analysis of calcium propionate

SEM revealed distinct differences in the surface morphology of calcium propionate synthesized from the four calcium sources (Figure 1). Calcium propionate synthesized from shellfish meal (Figure 1A) consisted of relatively large plate-like particles with smooth surfaces and compact aggregates. In contrast, the bone meal-derived product (Figure 1B) exhibited densely packed irregular granules with rough, heterogeneous surfaces, indicating a less uniform particle distribution. The eggshell flour-derived product (Figure 1C) displayed a highly porous and irregular microstructure characterized by interconnected cavities and loosely aggregated particles, suggesting a substantially greater apparent surface area than the other calcium sources. Meanwhile, calcium propionate synthesized from indigenous limestone flour (Figure 1D) exhibited layered, fractured particles with a relatively compact morphology and lower apparent porosity than the eggshell-derived product.

Among the evaluated calcium sources, eggshell flour produced calcium propionate with the most porous and heterogeneous morphology. The interconnected pore network and rough surface texture indicate a greater specific surface area, which may facilitate enhanced contact with aqueous media and improve dissolution characteristics. Such morphological properties are advantageous for feed additive applications because they can increase the release and bioavailability of calcium and propionate ions following ingestion. Similar observations have been reported for porous calcium-based materials, which exhibit improved physicochemical characteristics and faster dissolution than dense crystalline structures [14].

The marked differences in particle morphology demonstrate that the calcium precursor strongly influenced the microstructure of the synthesized calcium propionate. Eggshell flour consists predominantly of highly purified calcite (approximately 94%–97% calcium carbonate) with only minor quantities of organic matrix and trace minerals. This relatively homogeneous composition promotes a more complete neutralization reaction with propionic acid, resulting in fewer residual mineral phases and a more open microstructure [15]. In contrast, shellfish meal and bone meal contain appreciable amounts of phosphate, magnesium, silica, and other inorganic constituents that may interfere with crystal formation and promote heterogeneous particle aggregation. Mahmood *et al.* [14] similarly reported that calcium propionate synthesized from high-purity calcium carbonate exhibited greater structural uniformity and fewer morphological defects than products derived from less purified calcium sources.

The porous morphology of the eggshell-derived product may also provide important functional advantages in livestock nutrition. Materials with higher surface roughness and interconnected pores generally possess greater specific surface area, facilitating rapid hydration and dissolution. Improved dissolution enhances the availability of calcium and propionate ions, which contribute to calcium homeostasis and provide glucogenic substrates during periods of increased metabolic demand in ruminants [16]. Similar relationships between particle morphology, surface area, dissolution kinetics, and bioavailability have been widely documented for calcium carbonate biomaterials derived from natural waste resources [14, 17]. Therefore, the porous microstructure of eggshell-derived calcium propionate is expected to improve its physicochemical performance following incorporation into animal feed.

The SEM findings were corroborated by subsequent FTIR and XRD analyses, which confirmed successful calcium propionate synthesis and showed that the eggshell-derived product had greater chemical homogeneity and crystallinity than products synthesized from the other calcium sources. The agreement among morphological, structural, and crystallographic analyses indicates that eggshell flour is the most suitable calcium precursor for producing high-quality calcium propionate. Moreover, using eggshell waste provides an environmentally sustainable strategy for converting an abundant agro-industrial by-product into a value-added feed additive while supporting resource recycling and circular bioeconomy principles [14, 18].

**Figure 1:**
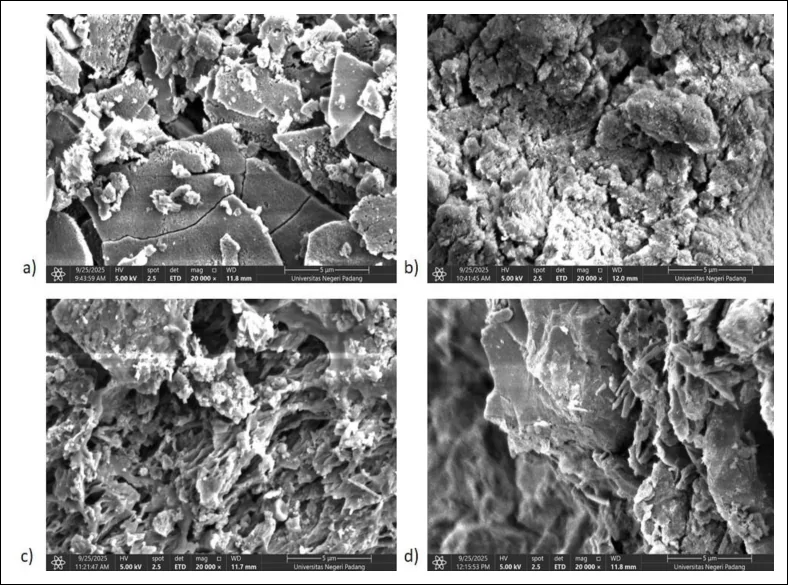
Scanning electron microscopy micrographs of calcium propionate synthesized from different calcium sources: (A) Shellfish meal, (B) Bone meal, (C) Eggshell flour, and (D) Indigenous limestone flour.

### Elemental composition analysis (EDS)

EDS was performed to determine the elemental composition of calcium propionate synthesized from the four calcium sources (Figure 2). The spectra confirmed that calcium, carbon, and oxygen were the predominant elements in all synthesized products, consistent with the expected composition of calcium propionate. However, noticeable differences in elemental purity and the presence of minor inorganic constituents were observed among the treatments, reflecting differences in the mineral composition of the precursor materials.

**Figure 2:**
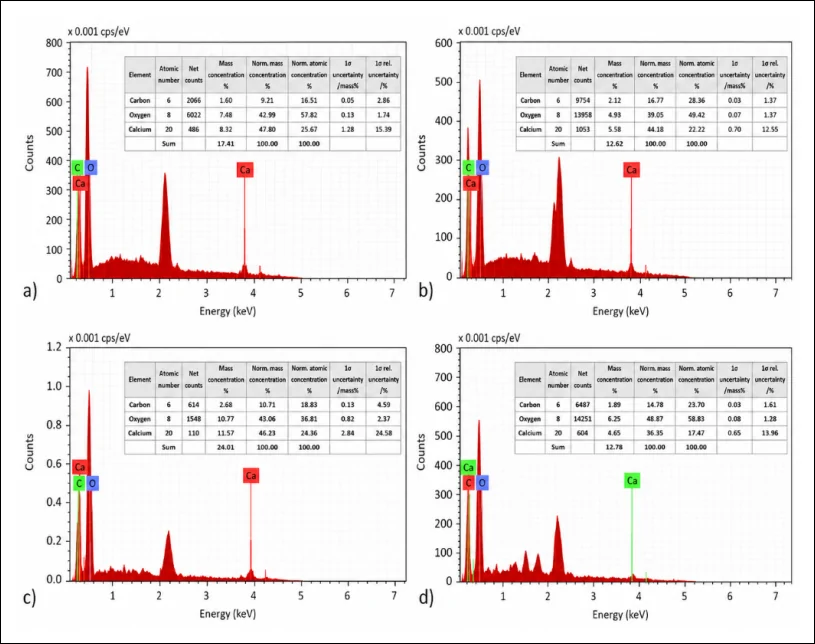
Elemental composition analysis spectra of calcium propionate synthesized from different calcium sources: (A) Shellfish meal, (B) Bone meal, (C) Eggshell flour, and (D) Indigenous limestone flour.

The eggshell flour-derived product exhibited the most homogeneous elemental composition, with calcium, carbon, and oxygen accounting for most detected elements and relatively few detectable impurities. This finding is consistent with the high calcium carbonate content of eggshells and supports the formation of a chemically uniform product. In contrast, products synthesized from shellfish meal, bone meal, and indigenous limestone contained additional minor elements, including phosphorus, magnesium, silicon, and other inorganic constituents originating from the respective raw materials. The persistence of these elements indicates incomplete removal of naturally occurring mineral impurities during synthesis and is consistent with previous observations for alternative calcium sources [14].

The higher elemental purity observed in the eggshell-derived calcium propionate supports the SEM findings, which demonstrated a more porous and homogeneous microstructure. Together, these results indicate that the relatively pure calcite composition of eggshell flour promoted a more efficient neutralization reaction with bio-derived propionic acid, thereby minimizing the incorporation of secondary mineral phases into the final product. Mahmood *et al.* [14] similarly reported that calcium propionate synthesized from high-purity calcium carbonate sources exhibited greater chemical uniformity and fewer structural impurities than products derived from less refined calcium precursors.

From a functional perspective, the combination of higher elemental purity and porous morphology may improve dissolution efficiency and the subsequent release of calcium and propionate ions following ingestion. Enhanced dissolution characteristics are desirable for livestock feed additives because they improve mineral availability and facilitate the rapid utilization of propionate as a glucogenic precursor during periods of increased metabolic demand [19–21].

The EDS findings complement the SEM, FTIR, and XRD analyses, collectively demonstrating that eggshell flour provides the most suitable calcium precursor for synthesizing high-quality calcium propionate. In addition to improving product quality, using eggshell waste offers an environmentally sustainable approach that converts an abundant agro-industrial by-product into a value-added feed additive while reducing dependence on commercially refined calcium sources and supporting circular bioeconomy strategies [22].

## FTIR

FTIR confirmed the successful synthesis of calcium propionate from all four calcium sources through the presence of characteristic absorption bands corresponding to propionate functional groups (Figure 3). Although all treatments exhibited similar spectral profiles, slight differences in peak intensity and sharpness were evident. The most prominent absorption band was observed at approximately 1400–1450 cm⁻¹, corresponding to the asymmetric stretching vibration of the carboxylate (COO⁻) group, which is the characteristic functional group of calcium propionate. Additional absorption bands within the 950–1100 cm⁻¹ region were attributed to C–O stretching vibrations of the propionate moiety, whereas weak bands between 2850 and 2950 cm⁻¹ corresponded to aliphatic C–H stretching vibrations. Broad absorption bands at 3200–3600 cm⁻¹ were assigned to O–H stretching of adsorbed moisture or residual hydroxyl groups commonly present in calcium-containing materials. These characteristic absorption bands agree with previously reported FTIR spectra of calcium propionate and confirm successful synthesis of the target compound [9, 23].

**Figure 3:**
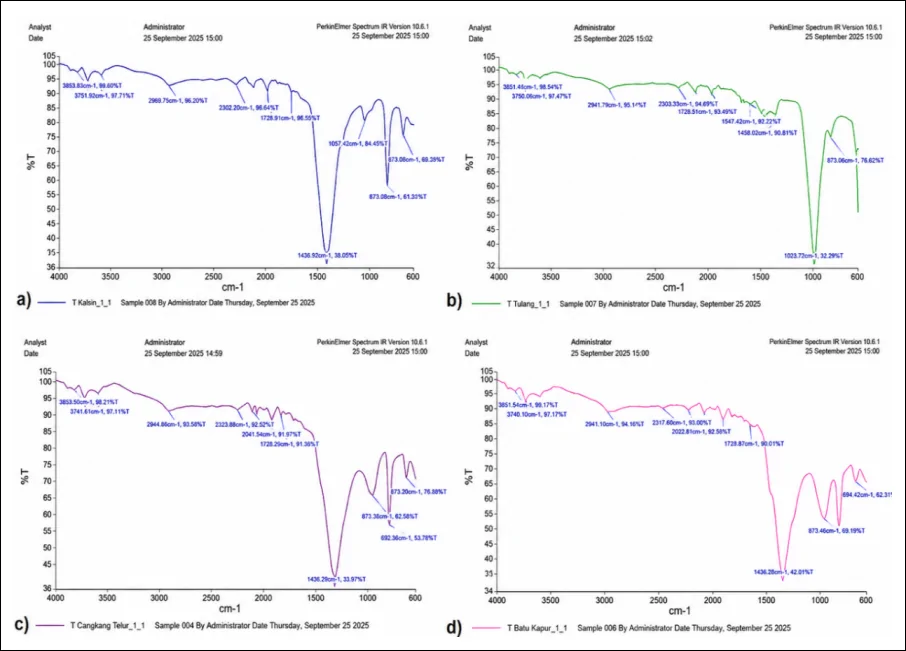
FTIR spectra of calcium propionate synthesized from different calcium sources: (A) Shellfish meal, (B) Bone meal, (C) Eggshell flour, and (D) Indigenous limestone flour.

Among the evaluated calcium sources, calcium propionate synthesized from eggshell flour showed the sharpest, best-defined absorption bands, indicating a more homogeneous chemical environment and reduced interference from secondary mineral phases or residual reactants. Eggshell flour consists predominantly of highly crystalline calcite with only minor amounts of magnesium carbonate, calcium phosphate, and organic matrix proteins [22]. Consequently, this high-purity calcium precursor promoted a more complete neutralization reaction with propionic acid, resulting in calcium propionate with fewer structural irregularities. In contrast, shellfish meal, bone meal, and indigenous limestone contained varying proportions of phosphate minerals, silica, magnesium, and other inorganic constituents that likely contributed to broader absorption bands through residual impurities remaining after synthesis. Similar findings have been reported for calcium salts synthesized from high-purity calcium carbonate sources [24].

The FTIR results agree closely with the SEM, EDS, and XRD analyses, collectively demonstrating that the calcium precursor substantially influenced the structural characteristics of the synthesized calcium propionate. The porous morphology observed by SEM, the relatively homogeneous elemental composition identified by EDS, and the well-defined FTIR absorption bands obtained from the eggshell-derived product consistently indicate a more complete synthesis process and a chemically homogeneous material. Furthermore, the close agreement between FTIR and XRD confirms that the synthesized product possessed the expected molecular structure and crystalline characteristics of calcium propionate.

Overall, these findings demonstrate that eggshell flour is the most suitable alternative calcium source for producing high-quality calcium propionate. In addition to improving product quality, using eggshell waste provides an environmentally sustainable strategy for producing value-added feed additives, reducing dependence on commercially refined calcium carbonate, and promoting circular bioeconomy principles through agro-industrial waste valorization [22, 25].

### Crystallinity analysis

XRD analysis showed that the crystalline characteristics of the synthesized calcium propionate varied with the calcium precursor used during synthesis (Figure 4). All diffraction patterns exhibited the characteristic reflections of calcium-containing crystalline phases, confirming successful conversion of bio-derived propionic acid into calcium propionate. However, the four treatments showed distinct differences in peak sharpness, intensity, and phase complexity. Calcium propionate synthesized from eggshell flour (Figure 4C) exhibited sharp, well-resolved diffraction peaks with relatively low background intensity, indicating a more ordered crystalline structure and greater phase homogeneity. In contrast, products synthesized from bone meal (Figure 4B) and indigenous limestone flour (Figure 4D) displayed broader, more complex diffraction patterns with additional reflections attributable to phosphate-, magnesium-, and silica-containing mineral phases, reflecting the heterogeneous mineral composition of these calcium sources. The shellfish meal-derived product (Figure 4A) exhibited an intermediate diffraction pattern, with greater crystallinity than bone meal but lower structural uniformity than the eggshell-derived product.

**Figure 4:**
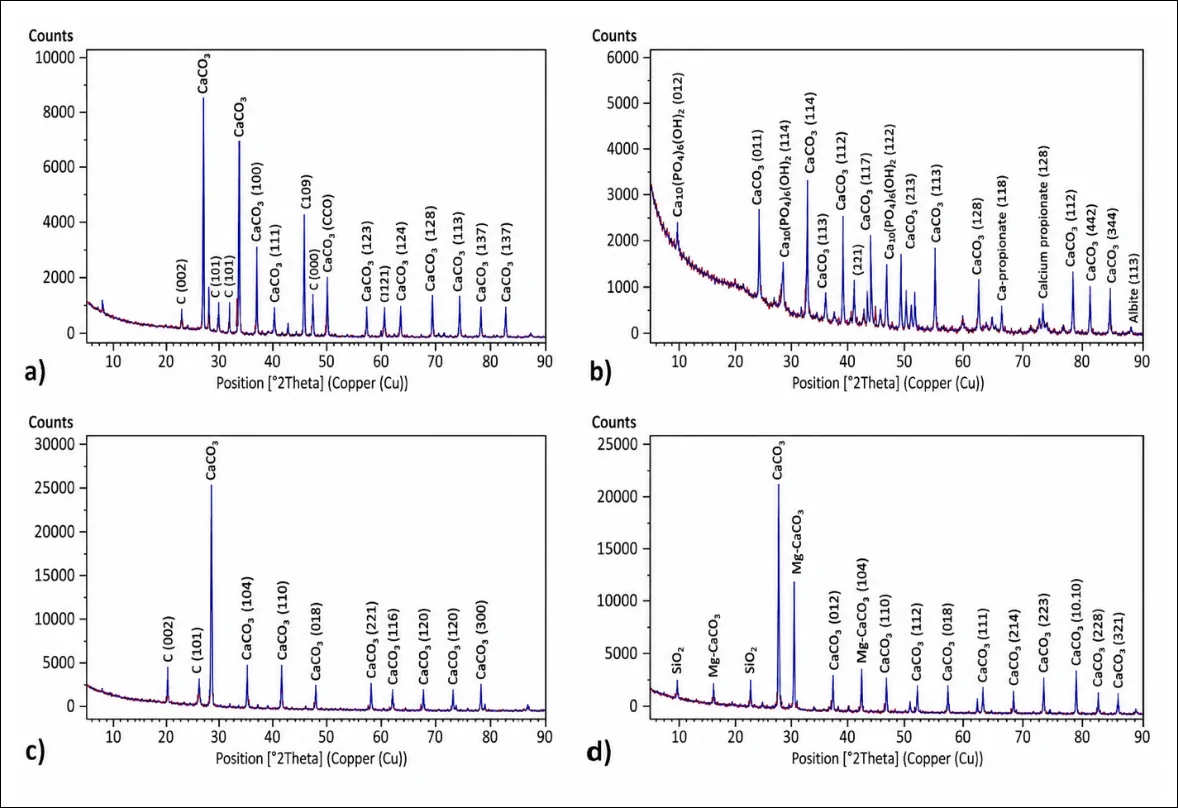
X-ray diffraction patterns of calcium propionate synthesized from different calcium sources: (A) Shellfish meal, (B) Bone meal, (C) Eggshell flour, and (D) Indigenous limestone flour.

The superior crystallinity of the eggshell-derived calcium propionate is closely associated with the mineralogical composition of eggshells, which consist predominantly of highly crystalline calcite (CaCO₃) with only minor quantities of organic matrix and trace minerals [22]. The high purity of calcite provides readily available calcium that reacts efficiently with propionic acid, thereby minimizing the formation of residual secondary mineral phases during synthesis. Conversely, bone meal naturally contains hydroxyapatite [Ca₁₀(PO₄)₆(OH)₂], whereas indigenous limestone commonly contains dolomite, silica, magnesium carbonate, and other accessory minerals that contribute to more complex diffraction profiles and indicate incomplete conversion of the precursor minerals. Mahmood *et al.* [14] similarly demonstrated that calcium propionate synthesized from high-purity calcium carbonate exhibited sharper diffraction peaks and fewer crystalline impurities than products prepared from heterogeneous calcium sources. Likewise, recent investigations of waste-derived calcium materials have shown that precursor purity is a major determinant of crystallinity, crystal growth, and structural homogeneity in synthesized calcium salts [26].

The greater crystallinity observed in the eggshell-derived product is likely to enhance its physicochemical stability during storage and processing. Well-ordered crystalline materials generally exhibit greater structural uniformity and reduced structural defects, contributing to improved product consistency and handling characteristics. Although excessive crystallinity may reduce dissolution in some mineral systems, the present findings indicate that the highly porous morphology observed by SEM compensated for this effect by providing an extensive surface area for hydration. Consequently, the eggshell-derived calcium propionate combined favorable crystallinity with a porous microstructure, characteristics desirable for feed additive applications requiring both storage stability and efficient dissolution after ingestion.

The XRD findings complement the SEM, EDS, and FTIR results, collectively demonstrating that eggshell flour produced calcium propionate with the most favorable morphological, elemental, chemical, and crystallographic characteristics among the evaluated calcium sources. The close agreement among these complementary analytical techniques provides strong evidence that eggshell flour is the most suitable alternative calcium precursor for synthesizing high-quality calcium propionate from bio-derived propionic acid. Furthermore, valorization of eggshell waste provides an environmentally sustainable strategy for converting an abundant agro-industrial by-product into a value-added feed additive while reducing reliance on commercially refined calcium sources and supporting circular bioeconomy principles [18, 22, 27].

### Implications for feed additive application

The present study demonstrates that calcium propionate synthesized from bio-derived propionic acid possesses physicochemical characteristics that support its potential application as a livestock feed additive. Complementary findings from SEM, EDS, FTIR, and XRD confirmed successful synthesis of calcium propionate and showed that the calcium precursor substantially influenced product quality. Among the evaluated calcium sources, eggshell flour consistently produced calcium propionate with the most favorable combination of porous morphology, relatively homogeneous elemental composition, well-defined functional groups, and highly ordered crystalline structure. Together, these characteristics are expected to improve product stability while facilitating rapid hydration and dissolution, thereby enhancing the release and availability of calcium and propionate ions within the gastrointestinal tract. Previous studies have shown that calcium propionate readily dissociates into calcium and propionate, enabling simultaneous mineral supplementation and provision of glucogenic substrates in ruminants [2, 28].

From a nutritional perspective, propionate is the principal precursor for hepatic gluconeogenesis in ruminants and contributes substantially to endogenous glucose production, particularly during periods of increased metabolic demand such as early lactation [6]. Adequate glucose availability is essential for maintaining milk production, supporting immune competence, and minimizing metabolic disorders associated with NEB. Dietary supplementation with calcium propionate has been shown to improve dry matter intake, feed efficiency, milk yield, and metabolic status while reducing the incidence of hypocalcemia, ketosis, and other transition-related disorders in dairy animals [1, 2, 28]. Although the present investigation focused exclusively on physicochemical characterization, the favorable structural properties of the eggshell-derived product suggest considerable potential for comparable biological performance. Nevertheless, controlled *in vivo* feeding trials are required to verify its bioavailability, safety, digestibility, and productive responses under commercial livestock production systems.

Beyond its nutritional advantages, utilizing eggshell waste as the primary calcium source provides substantial environmental and economic benefits. Eggshells are generated in large quantities by the food processing industry and are frequently discarded despite containing approximately 94%–97% calcium carbonate, making them an abundant and renewable calcium resource [22]. Converting this agro-industrial by-product into calcium propionate represents an effective waste valorization strategy that improves resource efficiency while reducing dependence on commercially refined calcium carbonate. Integration of bio-derived propionic acid production with eggshell recycling therefore represents a sustainable manufacturing approach that supports circular bioeconomy principles and offers a promising pathway for producing environmentally friendly, value-added feed additives for future livestock production systems [18].

## CONCLUSION

This study successfully demonstrated the sustainable synthesis of calcium propionate from bio-derived propionic acid produced by a co-culture of *S.* *harbinensis* and *P.* *kudriavzevii* using four alternative calcium sources. Comprehensive physicochemical characterization demonstrated that the calcium precursor substantially influenced product quality. Among the evaluated materials, eggshell flour consistently produced calcium propionate with the most favorable characteristics, including a highly porous surface morphology observed by SEM, a relatively homogeneous elemental composition determined by EDS, well-defined functional groups confirmed by FTIR, and a more ordered crystalline structure revealed by XRD. Collectively, these complementary analyses indicate that the high mineral purity of eggshell flour promoted more efficient synthesis and yielded calcium propionate with superior structural and physicochemical properties compared with shellfish meal, bone meal, and indigenous limestone flour.

The findings have important practical implications for sustainable livestock production. Integrating bio-derived propionic acid with eggshell waste provides an environmentally friendly alternative to conventional petrochemical-based calcium propionate production while valorizing an abundant agro-industrial by-product. The favorable physicochemical characteristics of the eggshell-derived product suggest improved dissolution behavior and the potential for efficient release of calcium and propionate following ingestion, supporting its prospective application as a functional livestock feed additive.

A major strength of this study is the successful integration of renewable microbial fermentation with waste-derived calcium utilization, supported by comprehensive characterization using complementary analytical techniques that consistently confirmed product quality. This integrated approach provides a scientifically robust framework for developing sustainable feed additives while advancing resource recycling and circular bioeconomy principles.

Nevertheless, several limitations should be acknowledged. This investigation focused exclusively on physicochemical characterization and did not quantify calcium propionate yield, reaction efficiency, dissolution kinetics, storage stability, or long-term product stability. Furthermore, biological efficacy, digestibility, bioavailability, and safety were not evaluated under *in vivo* conditions. Economic feasibility, process scalability, and life-cycle environmental impacts also remain to be established before commercial implementation.

Future studies should optimize large-scale fermentation and synthesis processes, quantify production efficiency and product purity, investigate dissolution kinetics and storage stability, and conduct controlled *in vivo* feeding trials to evaluate bioavailability, animal performance, metabolic responses, and safety. In addition, techno-economic analyses and life-cycle assessments are required to determine the commercial viability and environmental sustainability of this integrated production system.

In conclusion, eggshell flour represents the most suitable alternative calcium source for synthesizing high-quality calcium propionate from bio-derived propionic acid produced by *S.* *harbinensis* and *P.* *kudriavzevii*. Integrating microbial postbiotic production with eggshell waste valorization provides a sustainable, environ-mentally responsible strategy for manufacturing value-added feed additives. This approach has considerable potential to reduce reliance on petrochemical-derived raw materials, enhance resource utilization, support circular bioeconomy principles, and contribute to more sustainable livestock production systems.

## DATA AVAILABILITY

The datasets used for this study are available from the corresponding author upon reasonable request.

## GENERATIVE AI DECLARATION

Artificial intelligence tools were used solely to assist with language editing and manuscript organization. All generated content was carefully reviewed, verified, and revised by the authors, who accept full responsibility for the accuracy, integrity, and originality of the manuscript.

## AUTHORS’ CONTRIBUTIONS

YM, FA, HH, and LA: Conceptualization and study design. YM, LA, HH, LRA, and TAB: Preparation of materials, data collection, and data analysis. YM and HH: Methodology. LRA and TAB: Formal analysis and investigation. YM and LA: Writing of the original draft. YM, HH, LRA, and TAB: Manuscript review and editing. All authors have read and approved the final version of the manuscript.
